# Novel mitochondrion‐targeting copper(II) complex induces HK2 malfunction and inhibits glycolysis via Drp1‐mediating mitophagy in HCC

**DOI:** 10.1111/jcmm.14971

**Published:** 2020-01-28

**Authors:** Mengmeng Li, Jiangjuan Shao, Zijian Guo, Chun Jin, Ling Wang, Feixia Wang, Yan Jia, Zhenzhu Zhu, Ziji Zhang, Feng Zhang, Shizhong Zheng, Xiaoyong Wang

**Affiliations:** ^1^ State Key Laboratory of Coordination Chemistry School of Chemistry and Chemical Engineering Nanjing University Nanjing China; ^2^ Jiangsu Key Laboratory for Pharmacology and Safety Evaluation of Chinese Materia Medica School of Pharmacy Nanjing University of Chinese Medicine Nanjing China; ^3^ State Key Laboratory of Pharmaceutical Biotechnology School of Life Sciences Nanjing University Nanjing China; ^4^ Department of Pharmaceutical Technology Xuzhou Pharmaceutical Vocational College Xuzhou China; ^5^ School of Food Science and Engineering Nanjing University Of Finance & Economics Nanjing China

**Keywords:** copper complex, dynamin‐related protein 1, glycolysis, hexokinase 2, mitophagy

## Abstract

[Cu(ttpy‐tpp)Br_2_]Br (abbreviated as CTB) is a novel mitochondrion‐targeting copper(II) complex synthesized by our research group, which contains tri‐phenyl‐phosphonium (TPP) groups as its lipophilic property. In this study, we explored how CTB affects mitochondrial functions and exerts its anti‐tumour activity. Multiple functional and molecular analyses including Seahorse XF Bioanalyzer Platform, Western blot, immunofluorescence analysis, co‐immunoprecipitation and transmission electron microscopy were used to elucidate the underlying mechanisms. Human hepatoma cells were subcutaneously injected into right armpit of male nude mice for evaluating the effects of CTB in vivo. We discovered that CTB inhibited aerobic glycolysis and cell acidification by impairing the activity of HK2 in hepatoma cells, accompanied by dissociation of HK2 from mitochondria. The modification of HK2 not only led to the complete dissipation of mitochondrial membrane potential (MMP) but also promoted the opening of mitochondrial permeability transition pore (mPTP), contributing to the activation of mitophagy. In addition, CTB co‐ordinately promoted dynamin‐related protein 1 (Drp1) recruitment in mitochondria to induce mitochondrial fission. Our findings established a previously unrecognized role for copper complex in aerobic glycolysis of tumour cells, revealing the interaction between mitochondrial HK2‐mediated mitophagy and Drp1‐regulated mitochondrial fission.

## 1 INTRODUCTION

Hepatocellular carcinoma (HCC) is one of the primary malignant tumours with high mortality in the world. The diagnosis of HCC is not difficult, but its treatment cannot produce better expected results. It has been a primary task to study the key molecular mechanisms of HCC development for effective treatment.^1^, ^2^ In 1920, German biochemist Warburg discovered that the glycolytic activity of liver cancer cells is more active than normal liver cells. Studies on HCC metabolomics have shown that compared with paracancerous tissues, liver cancer tissues have higher glucose metabolism rate and glycolysis capacity four times that of oxidative phosphorylation.^3^ It is proposed that even under sufficient oxygen, the growth of malignant tumour cells is still dependent on glycolysis, exhibiting high glucose uptake rate and lactic acid content of metabolites to provide a variety of precursors for the essential nutrients and sufficient ATP.^4^

Hexokinase is the first rate‐limiting enzyme in the glycolytic pathway, catalysing the production of glucose‐6‐phosphate from glucose.^5^, ^6^, ^7^ In normal cells, hexokinase isozymes have low transcriptional expression levels and each has specific tissue specificity.^5^, ^7^ High expression of the mitochondrial‐binding hexokinase subtype HK2 is involved in the molecular basis of high glucose glycolysis rates in tumour cells.^8^ Compared to the other three subtypes, HK2 has higher affinity for certain proteins or protein channels, making it easier to "dock" on the mitochondrial outer membrane, via its binding to voltage‐dependent anion channels (VDAC).^9^, ^10^ This feature of HK2 in tumour cells is attracting more and more attention from researchers. The idea that HK2 is highly expressed in tumour cells just to ensure energy supply has begun to change. In tumour cells, mitochondrial HK2 not only promotes a aerobic glycolysis, but also increases resistance to cell death signals.^11^ Thus, the increased HK2 expression and its binding to mitochondria facilitates not only increased aerobic glycolysis and lactate production but also the channelling of glycolytic substrates into biosynthetic pathways for which mitochondria play a crucial role, so it seems that understanding the relationship between mitochondrion and glycolysis is important to explore how it performs related functions.

Autophagy is generally non‐selective cellular process that uses lysosomes to degrade its own damaged organelles and macromolecules.^12^ In addition to its role in normal physiology, autophagy plays an important role in the pathology of the body, and a large number of studies have explored the complex role of autophagy in cancer.^12^, ^13^ Mitophagy is a characteristic selection process regulated by various factors, containing PINK1/Parkin‐mediated pathway and NIX/BNIP3‐mediated signal pathway.^14^ Under normal conditions, mitochondria have a low membrane voltage, and then, PINK1 on the outer membrane of mitochondria can be rapidly degraded. In the case of damage to the mitochondria, the mitochondrial membrane is depolarized, and the voltage of the outer membrane is reduced. At this time, PINK1 cannot be immediately degraded, but stabilizes the outer membrane of the mitochondria and recruits Parkin to mitochondria.^15^ Parkin is an E3 ubiquitin ligase that can ubiquitinate mitochondrial proteins, such as VDAC1, forming a complex that cooperates with the kinesin‐like proteins to complete mitophagy.^16^ Mitochondria undergo constant renewal and their half‐life varies from tissue to tissue, which plays an active role in both physiological and pathological conditions.^17^ Tumour cells are particularly susceptible to the abnormalities of mitochondrial dynamics due to their high energy requirements, including mitochondrial fusion, fission and degradation, also known as mitophagy. Proteins with a role in mitochondrial dynamics are thus implicated in mitophagy to function together with the LC3 adapters/receptors. For example, dynamin‐related protein 1 (Drp1), a key motility protein that regulates mitochondrial fission, can interact with overexpressed FUNDC1. FUNDC1, an integral mitochondrial outer‐membrane protein, is a receptor for hypoxia‐induced mitophagy.^18^ Indeed, FUNDC1‐mediated mitophagy signal pathway may require Drp1 for mitochondrial dynamics.^19^, ^20^ In contrast, a recent report showed that mitophagy occurs without mitochondrial fission.^21^ Thus, the role of Drp1 and mitochondrial fission in mitophagy needs to be further addressed.

**Figure 1:**
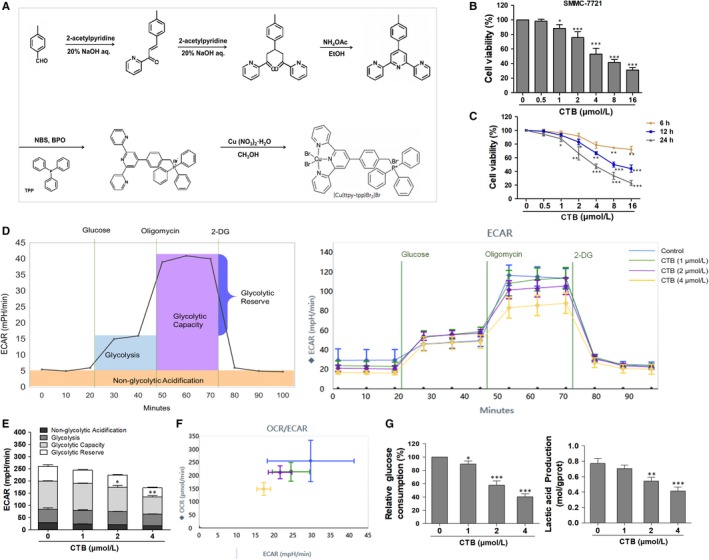
[Cu(ttpy‐tpp)Br_2_]Br (CTB) inhibited cells viability and repressed glycolysis in HCC cells. (A) The tripyridine ligand (ttpy) was synthesized first, and the TPP was introduced to synthesize the ligand ttpy‐tpp, and then, the coordination reaction with Cu (II) was carried out, and the product [Cu(ttpy‐tpp)Br_2_]Br was purified. (B) SMMC‐7721 cells were treated with concentrations of CTB (0, 0.5, 1, 2, 4, 8 and 16 μmol/L), and cells viability was determined by the MTT assay. (C) SMMC‐7721 cells were treated with concentrations of CTB (0, 0.5, 1, 2, 4, 8 and 16 μmol/L) at different time (6, 12, 24 h). (D) Measurement of extracellular acidification rate (ECAR) using the XFe24 Extracellular Flux Analyzer. (E) Glycolytic variations (glycolysis, glycolytic capacity and glycolytic reserve) were summarized from raw data. (F) Metabolic phenotypes were assessed by the ratio of OCR/ECAR. (G) Glucose consumption was detected using a glucose assay kit. Production of lactic acid was assayed by Lactic Acid Production Detection kit. Data were presented as mean ± SD (n = 5); significance: *P < .05, **P < .01 and ***P < .001 vs control

In this study, we cited a new copper complex, [Cu(ttpy‐tpp)Br_2_]Br (expressed as CTB, shown in Figure 1A), which is obtained by introducing tri‐phenyl‐phosphine (TPP) into copper‐terpyridine complex. TPP is a non‐toxic chemical group that exhibits mitochondrial targeting properties in living cells.^22^ TPP can impart delocalized charge and lipophilic properties to the compound, which facilitates mitochondrial accumulation.^23^ CTB has been found to have excellent anti‐tumour activity and mitochondrial targeting characteristic in our previous studies.^24^ However, the interaction between mitochondrial dysfunction and metabolic reprogramming in HCC cells with CTB treatment remains unclear. Therefore, the aim of this study was to reveal the interaction between mitochondrial physiological activity and glycolytic inhibition of hepatoma cells under CTB treatment.

## 2 MATERIALS AND METHODS

### 2.1 Reagents and antibodies

CTB ([Cu(ttpy‐tpp)Br_2_]Br) was synthesized in State Key Laboratory of Coordination Chemistry in Nanjing University. LDH test kit was purchased from Nanjing Jiancheng Bioengineering Institute (Nanjing, China). All antibodies were used at a dilution of 1:1000 unless otherwise specified according to the instructions. Antibodies to the following proteins HK2 (22029), PFKP (13389), PKM2 (15822) and VDAC1 (55529) were purchased from Proteintech. Antibodies to AKT (9272), p‐AKT (4060), Drp1 (8570), p‐Drp1 (Ser637) (4867), LC3A/B (12741), SQSTM1/p62 (23214), PINK1 (6946), Parkin (4211), β‐actin (3700) and COX IV (38563) were purchased from Cell Signaling Technology (Danvers, MA, USA). The XF24 Glycolysis Stress Test Kit (101706‐100), XF Cell Mito Stress Test Kit (102194‐100), XF Assay Medium (102353‐100) and XF Calibrant (100840‐000) were obtained from Agilent Seahorse (California, USA). L‐glutamine (G8540‐25G), glucose (G7528‐250G) and rapamycin (37094) were obtained from Sigma‐Aldrich (St Louis, MO, USA). 3‐Bromopyruvic acid (HY‐19992), Mdivi‐1(HY‐15886), was obtained from MedChem Express (New Jersey, USA).

### 2.2 Cell culture

Human hepatoma cells were purchased from Chinese Academy of Sciences (Beijing, China). The cells were cultured in RPMI 1640 (KeyGEN BioTECH, Nanjing, China) medium supplemented with 10% foetal calf serum (FBS; Gibco, Merelbeke, Belgium), 100 U/mL penicillin and 100 μg/mL streptomycin in incubator under the controlled condition of 95% air and 5% CO_2_ at 37ºC. Cell morphology was observed by Leica Qwin System. Prior to CTB treatment, cells grown to approximately 70%‐80% confluence and then were exposed to different concentrations (0‐4 μmol/L) of CTB for different time periods (0‐24 hours).

### 2.3 Animals and experimental procedures

All experimental procedures were approved by the Animal Care and Use Institutions and Local Committees of Nanjing University of Chinese Medicine (Nanjing, China), and all animals received humane care in accordance with the guidelines of the National Institutes of Health (USA). Four‐week‐old male nude mice (BALB/c‐nu/nu) weighing approximately 18‐20 g were procured from Nanjing Institute of Biomedical Research (Nanjing, China). All mice were housed in cages under sterile conditions to provide 12 hours of light‐dark circulation and sufficient water and food. To establish human HCC xenograft model, SMMC‐7721 cells were harvested at logarithmic phase, and 1 × 10^7^ cells/200 μL was subcutaneously injected into right armpit of each mouse to induce tumour growth. When the tumours had reached a mean size of 150 mm^3^, the tumour volumes were estimated every 3 days using callipers. The mice were randomly divided into seven groups (n = 8). Mice in Group 1 were served as a subcutaneous xenograft model. Mice in groups 2, 3 and 4 were served as treatment groups and i.p. injected by CTB with 5, 7.5 and 10 mg/kg, respectively. Mice in Group 5 were i.p. injected by 3‐BP (20 mg/kg). Mice in Group 6 were i.p. injected by Mdivi‐1 (50 mg/kg). Mice of Group 7 were served as a combined administration group and i.p. injected by CTB (7.5 mg/kg) and Mdivi‐1 (50 mg/kg). CTB was suspended in sterile PBS and injected three times a week, and the model group received the same volume of saline. Body weight was recorded every 3 days. Excision of parts of the tumour tissue was fixed in 4% paraformaldehyde for IHC/IF assay, 2.5% glutaraldehyde for transmission electron assay or frozen in liquid nitrogen.

### 2.4 Metabolic analyses

Glycolysis capacity (extracellular acidification rate, ECAR) and oxygen consumption rate (OCR) were measured using Seahorse Extracellular Flux (Seahorse Biosciences, XF‐24) analyser according to the manufacturer's protocol. Briefly, after the SMMC‐7721 cells grew well, 50 000 cells per well were plated into XF24 V7 PS Cell Culture Microplates (Seahorse Biosciences, 09516) and incubated for 12 hour at 37˚C in a 5% CO_2_ humidified atmosphere. Cells were washed in XF assay medium and were then kept in XF assay medium at 37˚C, in a non‐CO_2_ incubator for 1 hour. For OCR, 2 μmol/L oligomycin, 5 μmol/L FCCP and 2 μmol/L antimycin A and rotenone were loaded into the injection ports in the XFe 96 sensor cartridge in sequence. For ECAR, within the incubation time, 10 mmol/L glucose, 1 μmol/L oligomycin and 20 mmol/L 2‐DG were loaded into the injection ports in the XFe 96 sensor cartridge in sequence.

### 2.5 Glucose uptake measurement

The concentrations of glucose in the supernatant were measured and calculated using a kit (BioVision, K686) according to the manufacturer's instructions. Briefly, SMMC‐7721 cells were seeded in 6‐well plates. After the cells were attached, the cell culture medium was discarded and replaced with fresh medium containing different concentrations of CTB for 24 hours. The supernatant in the six‐well plate was collected. The absorbance was measured by using an Automatic Biochemical Analyzer (7170A, HITACHI, Tokyo, Japan). The relative glucose consumption rate was normalized by the protein concentration of the samples.

### 2.6 Detection of lactic acid content

Lactic acid is an important intermediate in the metabolism of organisms and is closely related to glucose metabolism and intracellular energy metabolism. Lactic acid content is an important indicator for assessing carbohydrate metabolism and aerobic metabolism. The concentrations of lactic acid in cells supernatant and tumour tissues were measured using a kit (BioVision, K627) according to the manufacturer's instructions.

SMMC‐7721 cells were seeded in 6‐well plates. After the cells were attached, the cell culture medium was discarded and replaced with fresh medium containing different concentrations of CTB for 24 hours. The supernatant in the six‐well plate was collected. For tumour tissue, the volume of the extract was determined according to the mass to volume ratio, homogenized in an ice bath and centrifuged at 12 000 g for 10 minutes at 4°C, and the supernatant was taken for measurement. The supernatant in the six‐well plate was collected. The absorbance was measured by using an Automatic Biochemical Analyzer (7170A, HITACHI, Tokyo, Japan).

### 2.7 Western blot analyses

Cells or tissue samples were lysed using mammalian lysis buffer (Sigma, St. Louis, MO, USA). BCA assay kit (Beyotime, China) measured the concentration of protein obtained. After blocking with 5% non‐fat dry milk for 2 hours, the membrane was incubated with specific primary antibody overnight at 4°C. After washing three times with TBS‐Tween‐20, the membrane was incubated with the secondary antibody for 2 hours at room temperature. Protein bands were visualized using a luminescent liquid (Millipore, USA). β‐actin was used as an invariant control for total protein and cytoplasmic proteins, and COX IV was used for mitochondrial proteins. The levels of target protein were densitometrically determined using Image Lab.

### 2.8 Analysis of autophagic flux

To determine autophagic flux, hepatoma cells were transfected with GFP‐tagged LC3B or tandem mRFP‐GFP‐tagged LC‐3 using Lipofectamine 2000 transfection reagent according to the manufacturer's instructions (Gene Chem). The transfected cells were then treated with CTB (1, 2, 4 μΜ), 3‐BP (50 μΜ) or Mdivi‐1 (50 μΜ) for 24 hours. Subsequently, these cells were fixed with 4% paraformaldehyde for 30 minutes. Random acquisition of GFP/mRFP images by laser scanning confocal microscopy (Olympus FV1000).

### 2.9 Hexokinase activity assay

Hexokinase activity was measured indirectly by the Hexokinase Activity Assay Kit (spectrophotometry; Beyotime, China), as a reading produced by NADPH. The cells were collected into a centrifuge tube, and the supernatant was discarded after centrifugation. According to the number of cells, the extract was formulated (500‐1000:1) and the cells were broken (ice bath) and centrifuged at 10 000 × g for 10 minutes at 4°C. The supernatant is collected and placed on ice for testing.

### 2.10 Immunoprecipitation assay

An immunoprecipitation assay was performed using extracts of the HCC cells as previously described.^25^ Briefly, immunoprecipitation was performed using Pierce cross‐linking magnetic IP/Co‐IP kit (Thermo Scientific) to analyse the interaction between HK2 and VDAC1.

### 2.11 Immunofluorescence analyses

Cells were seeded in 24‐well plates and treated with different reagents at indicated concentrations for 24 hours. To assess the subcellular localization of HK2/Drp1/Parkin (1:200 dilution), we used cells labelled with Mito‐Tracker Green (KGMP0072, Nanjing, China). To analyse the colocalization of lysosomes with mitochondria, we used cells double labelled with Lyto‐Tracker Red DND‐99 (40739ES50, Nanjing, China). Then, specific experimental methods refer to published literature.^25^

### 2.12 Detection of mitochondrial membrane potential

Mitochondrial membrane potential assay kit (Beyotime, China) with JC‐1 is an ideal probe widely used. When the MPP is high, JC‐1 aggregates in the mitochondrial matrix to form a polymer, which can produce red fluorescence; when the MPP is low, JC‐1 cannot aggregate in the mitochondrial matrix to form a monomer, which can produce green fluorescence. The cells were cultured in 24‐well plates (1 × 10^6^) after drug treatment. JC‐1 staining solution was added, and incubation was carried out for 20 minutes at 37°C in a 5% CO_2_ cell incubator. Laser microscopy detection: excitation wavelength 488‐505 nm, emission wavelength 575‐590 nm.

### 2.13 Rhodamine 123 staining

Rhodamine 123 is a cationic fluorescent dye that can penetrate cell membranes. When the integrity of the mitochondrial membrane is destroyed, Rh123 re‐releases the mitochondria, thereby emitting strong fluorescence. The cells were cultured in 24‐well plates (1 × 10^6^) after drug treatment. Rhodamine 123 staining solution (10 μg/mL) was added, and incubation was carried out for 30 minutes at 37°C in a 5% CO_2_ cell incubator. Laser microscopy detection: excitation wavelength 488‐505 nm, emission wavelength 515‐575 nm.

### 2.14 Transmission electron microscopy

Briefly, cells were seeded on a four‐compartment slide and fixed in 4% propylene glycol‐glutaraldehyde for 1 hour. Images were acquired randomly under different magnifications using a JEM‐1400 transmission electron microscope.

### 2.15 Statistical analyses

All the quantitative data were described as the mean ± standard deviation (SD) of at least three independent experiments and were analysed using GraphPad Prism 5 (San Diego, CA, USA). The significance of difference was determined by one‐way ANOVA with the post‐hoc Dunnett's test. Values of *P* < .05 were considered to be statistically significant.

## 3 RESULTS

### 3.1 [Cu(ttpy‐tpp)Br_2_]Br (CTB) inhibited cells viability and repressed glycolysis in HCC cells

The tripyridine copper(II) complex (which was selected as a prodrug) with high DNA cleavage activity in previous study was selected,^26^ and a TPP group with targeted mitochondrial function was introduced as a ligand.^24^ As shown in Figure 1A, the tripyridine ligand, 4′‐p‐tolyl‐2,2′:6,2″‐terpyridine (ttpy), was synthesized first. TPP was introduced into ttpy‐tpp and then co‐ordinated with copper (II) to obtain a product. TPP confers delocalized charge and lipophilic properties on the compound, favouring mitochondria accumulation (Figure S1A). Firstly, we investigated the anti‐tumour effect of CTB on different hepatoma cells and explored whether CTB was cytotoxic to LO2 cells. Cells were treated with CTB before cell viability assay, showing that a potent and dose/time‐dependent inhibition to the cells and the dosage range of CTB we chose to conduct the further study was 1‐4 μmol/L (Figure 1B, 1; Figure S1B).

To access whether mitochondrial dysfunction in HCC cells after CTB treatment was associated with the metabolic differences (potential Warburg effect), we conducted bioenergetics profiling of these cells using the Seahorse XF Bioanalyzer Platform. Tumour cells rely on high glycolysis rates to meet their rapid, uncontrolled proliferation and most of the energetic needs.^27^ We monitored the glycolytic activity of HCC cells through real‐time changes in ECAR levels. ECAR profiles showed that compared with the untreated cells, the basal extracellular acidification rate decreased in HCC cells treated with different doses of CTB (Figure 1D). After the addition of oligomycin, oxidative phosphorylation was inhibited, and the acid production increased. The increased value represented that the cell also had glycolysis ability, and ECAR profiles showed that CTB could inhibit this maximum potential. 2‐DG is a glycolysis inhibitor, and the value indicates that it is caused by a mechanism other than glycolysis. After treatment with CTB (4 μmol/L), the low glycolysis reserve capacity of the cells indicates that these cells operate at their maximum glycolysis rate and have the ability to increase their glycolysis flux in response to additional metabolic stress (Figure 1E). In summary, the ECAR profiles demonstrated the sensitivity of glycolysis to the effects of CTB.

Meanwhile, we also assessed the real‐time alterations of mitochondrial oxygen consumption rate (OCR). The basal level of OCR (at 0 minutes) was progressively decreased after replacement with the glucose‐free XF culture medium for 1 hour (Figure S1C). Upon treatment with oligomycin, there was less OCR reduction, indicating that CTB treatment had impaired the ability of mitochondria to utilize oxygen consumption for ATP production in HCC cells (Figure S1D). But based on our data, we have the effect of CTB on spare respiratory capacity. The OCR and ECAR levels of the above assessments were plotted as fold change relative to controls, so we observed the inhibition of maximal respiration was more obvious than the inhibition of glycolytic capacity by CTB (Figure 1F; Figure S1E). Conversion of glucose to lactic acid, glucose consumption and lactate production was measured to reflect glycolysis level. The results showed that CTB treatment led to a significant reduction in the levels of glucose consumption and the production of lactate in SMMC‐7721 cells (Figure 1G). Based on the above analysis, we initially determined the inhibitory effect of CTB on the glycolysis and metabolism of related substances in HCC cells.

### 3.2 CTB inhibited hepatoma cells glycolysis by inducing dissociation of HK2 from the mitochondria

**Figure 2:**
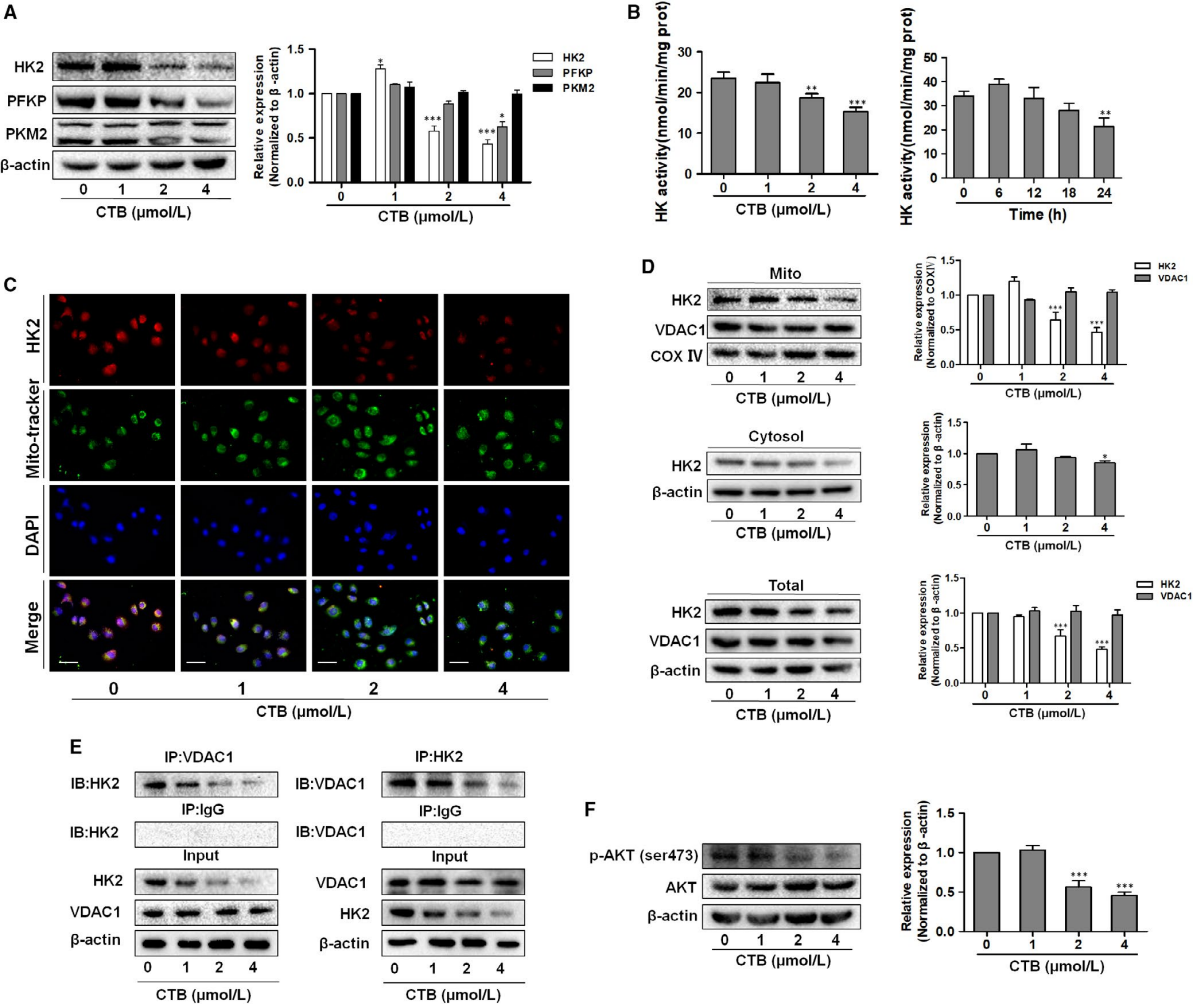
CTB inhibited the expression of HK2 and promoted dissociation of hexokinase 2 from the mitochondria. SMMC‐7721 cells were treated with indicated concentrations of CTB (0, 1, 2 and 4 μmol/L). (A) Protein levels of HK2, PFKP and PKM2 were measured by Western blot analysis. (B) HK activity was detected via Hexokinase Activity Detection Kit at different time (0, 6, 12, 18, 24 h). (C) Representative fluorescence microscope images of SMMC‐7721 cells labelled with DAPI, HK2 antibody and Mito‐Tracker Green. Scale bar: 50 μm. (D) Mitochondrial and cytosolic fractions were isolated and subjected to Western blot analysis for the expression of HK2. (E) Immunoprecipitation assay showed the interaction of HK2 and VDAC1. (F) Protein levels of p‐AKT and AKT were measured by Western blot analysis. Data were presented as mean ± SD (n = 3); significance: *P < .05, **P < .01 and ***P < .001 vs control

To further explore the mechanism of glycolysis suppression, we sought to identify possible target proteins for CTB responsible for regulating glycolysis. To verify which proteins in the glycolytic pathway are affected, we tested the levels of enzyme kinase, HK2, PKM2 and PFKP. We found that the degree of reduction in HK2 level was significantly higher than that of the other glycolytic enzymes (Figure 2A). We examined the mRNA levels corresponding to glycolytic proteins in cells transfected with CTB. We found that most of the genes encoding glycolytic proteins, including HK2, had similar levels of mRNA (Figure S2A). Therefore, CTB did not affect HK2 at the transcriptional level. Moreover, CTB inhibited the activity of hexokinase activity dose/time‐dependently in hepatoma cells (Figure 2B). Therefore, we hypothesized that HK2 may play a key role in the inhibition of glycolysis in hepatoma cells by CTB.

Studies have shown that HK2 has a stronger ability to bind to mitochondria in cancer cells, which may be more conducive to metabolic activity and survival.^10^, ^28^ Therefore, we determined whether the decrease of glycolysis had relationship on the localization of HK2 in hepatoma cells. The results in Figure 2C suggested lower levels of HK2 in response to CTB, Then, we examined the alteration of HK2 in mitochondrial fractions and cytoplasmic by Western blot. The expression of mitochondrial HK2 was decreased, while the level of HK2 in the cytoplasm was slightly decreased at high dose. These results indicated that CTB induced the isolation of mitochondrial HK2 and resulted in decrease in HK2 activity, but no significant effect on HK2 in cytoplasm (Figure 2D). VDAC, located in the mitochondrial outer membrane, functions as gatekeeper for the entry and exit of mitochondrial metabolites, thereby controlling cross‐talk between mitochondria and the rest of the cell.^29^ VDAC1 can be combined with the N‐terminal domain of HK2, allowing HK2 to attach to the outer membrane of the mitochondria.^30^, ^31^ The binding capacity of HK2 with VDAC1 diminished in a dose‐dependent manner by co‐immunoprecipitates analysis in cells treated with CTB (Figure 2E). Activated AKT plays a strong anti‐apoptotic role in tumour cells with high glycolysis rate, and the binding of HK2‐VDAC1 is regulated by upstream Akt.^32^ To assess whether CTB can affect the activation of the Akt signalling pathway, Western blot and immunofluorescence were used to determine the expression of p‐Akt and Akt. As shown in Figure 2F and Figure S2B, the expression of p‐Akt was decreased and the activation of Akt was inhibited after CTB treatment, reducing the sensitivity of binding of HK2 to VDAC1.

### 3.3 Mitophagy was activated by mPTP opening induced by HK2 separation from mitochondria

**Figure 3:**
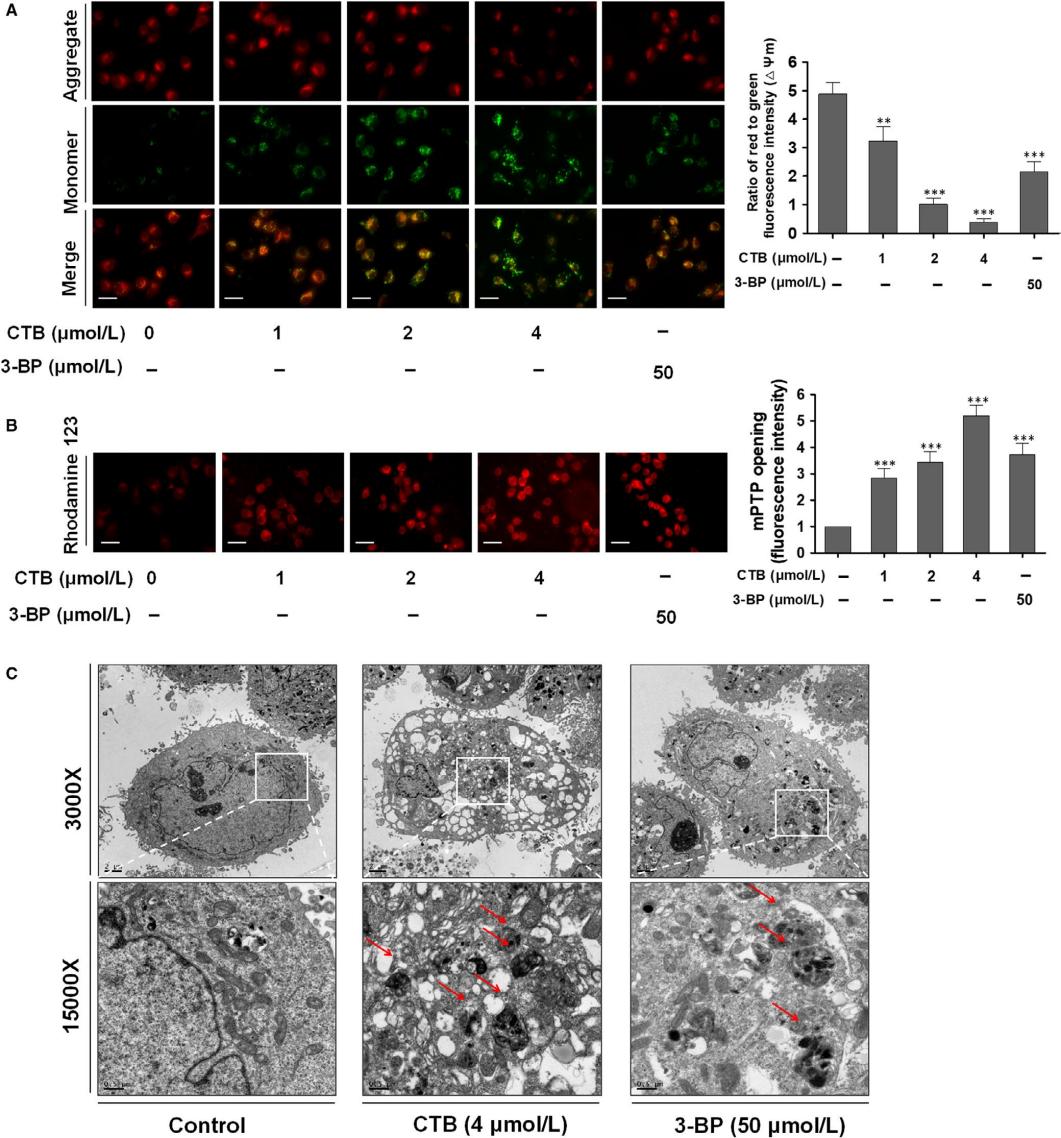
mPTP opening was induced by HK2 separation from mitochondria. SMMC‐7721 cells were treated with indicated concentrations of CTB (0, 1, 2 and 4 μmol/L) or 3‐BP (50 μmol/L) for 24 h. (A) The mitochondrial membrane potential (ΔΨm) was measured by JC‐1 staining. Scale bar: 50 μm. (B) mPTP opening was viewed by Rhodamine 123. Scale bar: 50 μm. (C) Observation of mitochondrial structure by transmission electron microscopy

At present, the composition and regulation of mPTP has not been fully understood and it may be regulated by the VDAC, the adenine nucleotide channel ANT and other cyclophilin.^33^ In order to determine the role of HK2 segregation in mPTP opening, 3‐bromopyruvate (3‐BP) was introduced, an inhibitor of mitochondrial bound hexokinase, as a positive control. We found that changes in the binding of HK2 to mitochondria not only led to the dissipation of ΔΨm, but also promoted the opening of mPTP (Figure 3A, 3). In order to determine the direct effect of CTB on mitochondria, we investigated the morphology of HCC cells by transmission electronic microscopy (TEM). We observed a significant accumulation of membrane vesicles containing subcellular material, indicating the presence of autophagy in CTB‐treated cells (red arrow in Figure 3C). Correspondingly, smaller autophagosomes were observed in 3‐BP‐treated cells compared to CTB treatment, indicating that mPTP opening, dissipation of ΔΨm and mitochondrial separation of HK2 did play a decisive role in activating autophagy pathways. Correspondingly, smaller autophagosomes were observed in 3‐BP‐treated cells, indicating that mitochondrial separation of HK2 did play a decisive role in activating autophagy pathways.

**Figure 4:**
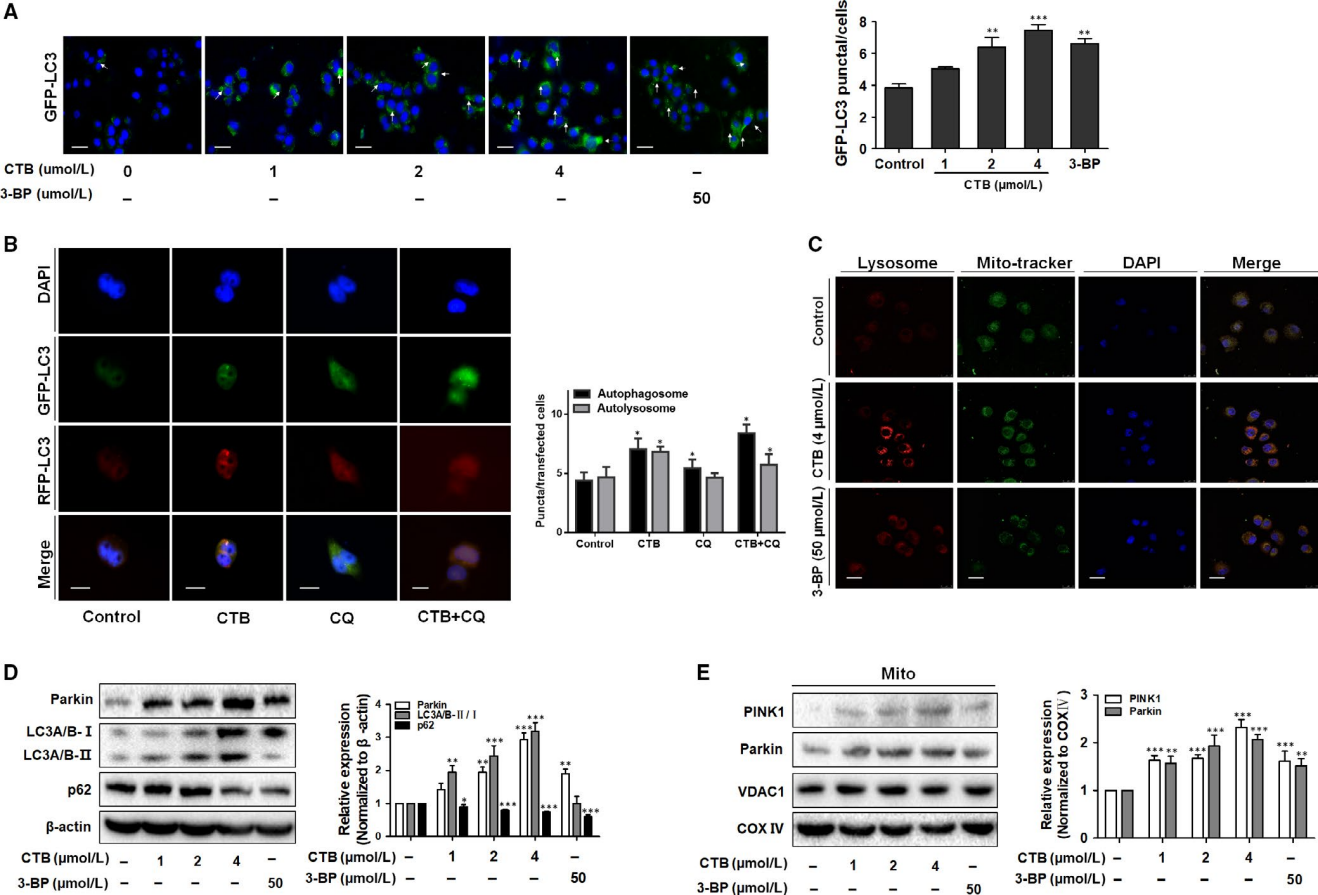
Mitophagy was activated by mPTP opening that induced by HK2 separation from mitochondria. (A) Cells expressing GFP‐LC3 were plated in complete media that was replaced with either CTB or 3‐BP for 24 h and analysed for LC3 dots. Scale bar: 100 μm. (B) Cells were transiently transfected with tandem fluorescent mRFP‐GFP‐tagged LC3 plasmid (RFP‐GFP‐LC3). Scale bar: 2 μm. (C) Representative images of colocalization of lysosomes and mitochondria using Mito‐Tracker Red and Lyso‐Tracker green staining. Scale bar: 20 μm. (D) Western blot analysis showed the protein expression of LC3A/B‐Ⅰ/Ⅱ and p62. (E) Western blot analysis showed the protein expression of Pakin and PINK1. Data were presented as mean ± SD (n = 3); significance: *P < .05, **P < .01 and ***P < .001 vs control

As further confirmation of the autophagic activity, we used GFP‐LC3B reporter to examine the recruitment of LC3 into autophagosomes. GFP‐LC3B fluorescence analysis showed that the number of GFP‐LC3B puncta profoundly increased upon CTB or 3‐BP treatment compared with that in cells cultured in normal conditions (Figure 4A). Due to the development of molecular biology, the mRFP‐GFP‐LC3 dual fluorescent autophagy indicator system has been used to label and track changes in LC3 and autophagic flux. Since the autophagosome enters the second stage, it fuses with the lysosome to form an autophagolysosome. As shown in Figure 4B, the results showed that the number of autophagosome and autolysosomes in CTB‐pretreated hepatocarcinoma cells was increased. After the combination of CTB and chloroquine (CQ, an autophagic late step inhibitor), the number of autolysosomes was decreased, suggesting that CQ impaired the promoting effect of CTB on autophagic flux.

In addition, colocalization of mitochondria and lysosomes demonstrates the presence of mitophagy (Figure 4C). CTB or 3‐BP reduced p62 levels, a receptor and substrate protein degraded by autophagy, increasing LC3A/B‐II levels and Parkin expression, a mitophagy‐associated protein, in HCC cells (Figure 4D). When mitochondria sense some induction signal, PINK1 will recruit to the mitochondrial membrane to activate Parkin, promoting the occurrence of mitophagy.^34^ The increased colocalization of Parkin and mitochondria explained that CTB activated Parkin and promoted its translocation on mitochondria, observed by immunofluorescence staining (Figure S3). In addition, CTB increased the content of PINK1 in mitochondria (Figure 4E), which is consistent with the transition from LC3‐I to LC3‐II. In conclusion, we speculated that CTB‐activated mitophagy may result in energy shortage and subsequent cellular death, playing a key role in anti‐tumour effects.

### 3.4 CTB induced mitochondrial fission via the regulation of Drp1, contributing to the mitophagy and disruption of mitochondrial structure

**Figure 5:**
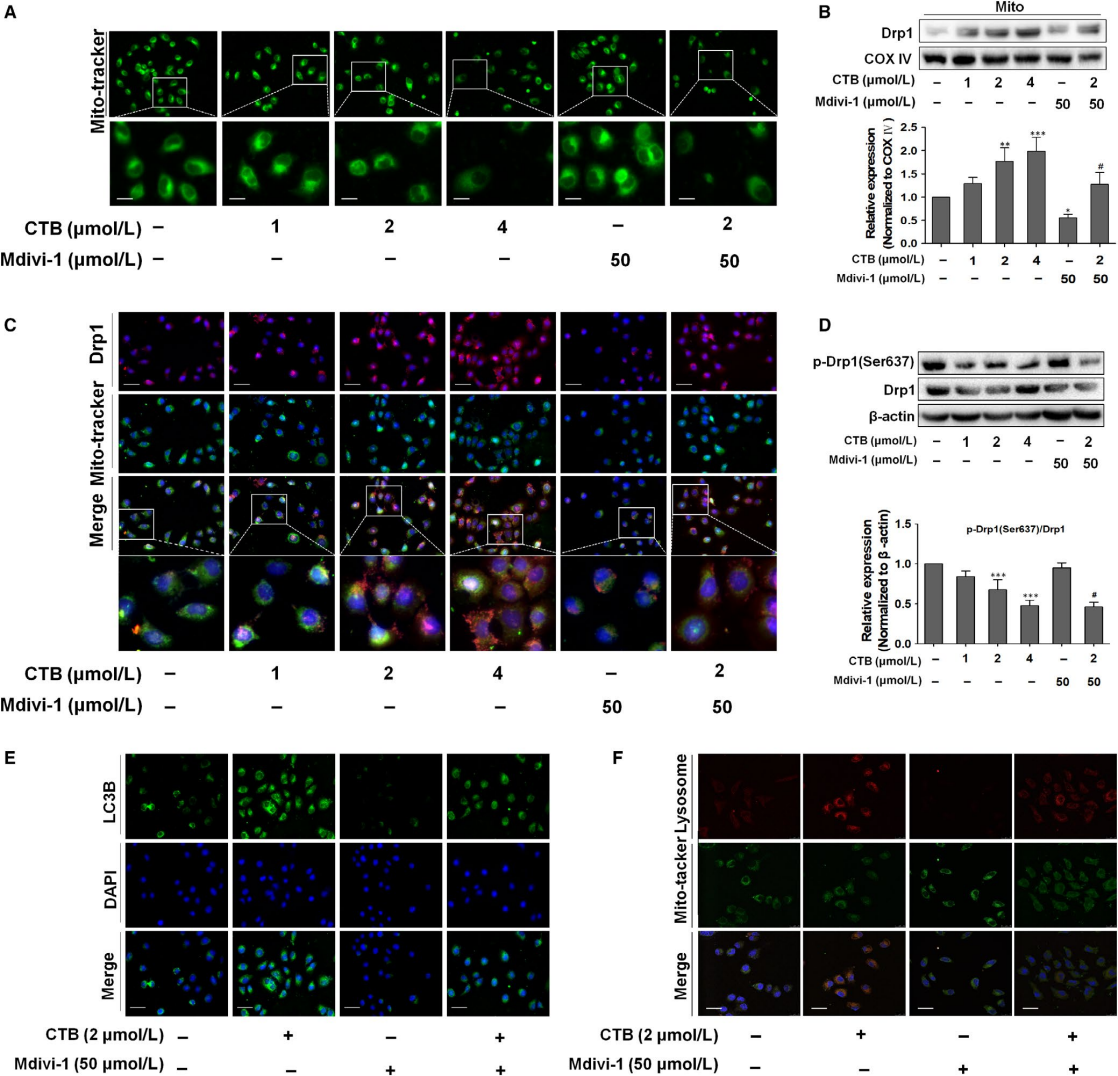
CTB induced mitochondrial fission via the regulation of Drp1, contributing to the mitophagy and disruption of mitochondrial structure. SMMC‐7721 cells were treated with indicated concentrations of CTB (0, 1, 2 and 4 μmol/L) or Mdivi‐1(50 μmol/L) for 24 h. (A) Mitochondrial fission was detected by Mito‐Tracker Green with fluorescence microscope. The boxed area under each micrograph was enlarged to determine mitochondria fragmentation. Scale bar: 10 μm. (B) Drp1 expression in mitochondria fraction was analysed by Western blot analysis. (C) Immunostaining showed the activation and location of Drp1 in SMMC‐7721 cells labelled with DAPI, Drp1 antibody and Mito‐Tracker Green. Scale bar: 50 μm. (D) Protein levels of p‐Drp1 and Drp1 were measured by Western blot analysis. Scale bar: 50 μm. (E) SMMC‐7721 cells expressing GFP‐LC3 were plated in complete media that was replaced with either CTB (4 μmol/L) or Mdivi‐1 (50 μmol/L) for 24 h and analysed for LC3 dots. Scale bar: 50 μm. (F) Representative images of colocalization of lysosomes and mitochondria. Scale bar: 25 μm. Data were presented as mean ± SD (n = 3); significance: *P < .05, **P < .01 and ***P < .001 vs control; ^#^ *P* < .05, ^##^ *P* < .01 and ^###^ *P* < .001 vs CTB treatment

To explore the reason for the induction of HK2 separation and mitophagy by CTB, we focus on mitochondrial fission. Mitochondrial fission proteins regulate membrane dynamics during various cellular events by self‐assembly.^35^ We examined the regulation of mitochondrial fusion‐related proteins, founding that CTB had less effect on mitochondrial fusion (Figure S4A). Then, we used Mdivi‐1, selectively inhibiting mitochondrial fission by blocking dynamin GTPase activity of Drp1, as the negative control group. First, we detected the LDH level in supernatant of hepatoma cells after Mdivi‐1 administration by kit to select the appropriate dose of Mdivi‐1 (Figure S4B). We observed that the mitochondria marked by Mito‐Tracker Green had greater amounts of free debris in CTB‐treated cells, while Mdivi‐1 weakened these effects (Figure 5A). We also detected loss of MMP and mPTP opening in the context of inhibition of mitochondrial fission (Figure S4C). Activation of the cytoplasmic Drp1 and aggregation of the mitochondrial surface were essential for mitochondrial fission.^36^ CTB significantly increased the overlap of Drp1 and mitochondria, while promoting the protein level of Drp1 in mitochondria (Figure 5B, 5). Drp1 activation and mitochondrial aggregation are involved in mitochondrial fission, whereas Drp1 activation can be inhibited by phosphorylation at Ser637.^37^ We found that CTB suppressed Drp1 phosphorylation (Ser637) in a dose‐ and time‐dependent manner to promote the activation of Drp1 (Figure 5D; Figure S4D), whereas Mdivi1 attenuates this effect.. These results indicated that CTB promoted mitochondrial fission by activating Drp1.

We further elucidated whether mitochondrial fission regulated mitophagy in HCC cells. Cells with Drp1 suppression had significantly less GFP‐LC3B dots than control cells, whereas CTB treatment had more accumulation of GFP‐LC3B dots (Figure 5E). Mdivi‐1 significantly reduced the expression level of LC3A/B‐II and increased the expression of p62 in HCC cells (Figure S4E). Systematically, the effect of CTB on the increased colocalization of mitochondria and lysosomes could be reversed by Mdivi‐1(Figure 5F). We also evaluated the mitophagic flux by detecting the expression of PINK1 and Parkin in mitochondrial fragmentation (Figure S4F), which provided further evidence supporting the presence of mitophagy. All these results supported the idea that the occurrence of mitophagy was closely dependent on the mitochondrial fission in CTB‐treated HCC cells.

### 3.5 The Drp1‐dependent fission and mitophagy facilitated HK2 separation from the mitochondria and the inhibition of glycolysis

**Figure 6:**
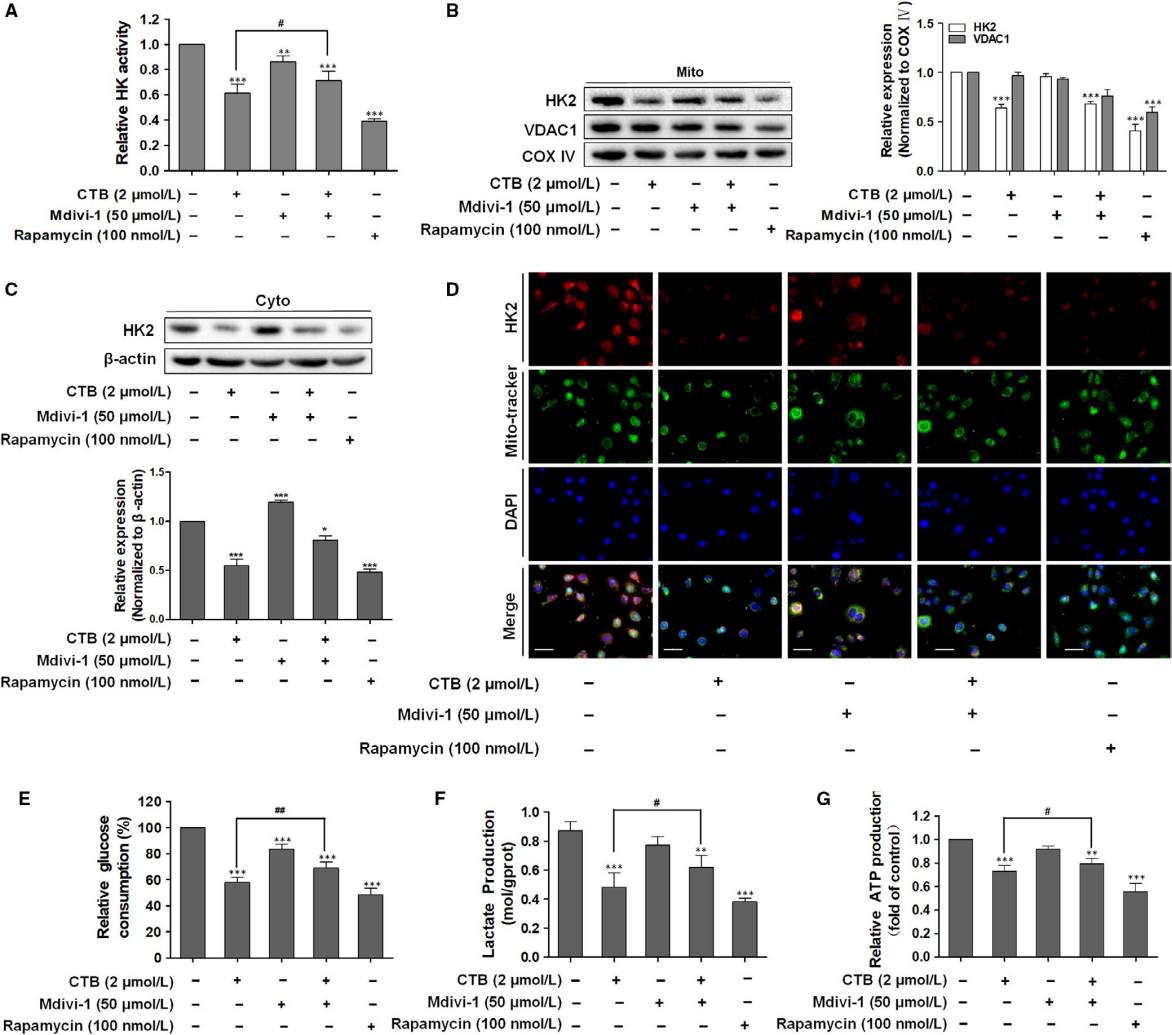
The Drp1‐dependent fission and mitophagy facilitated HK2 separation from the mitochondria and the inhibition of glycolysis. SMMC‐7721 cells were treated with indicated concentrations of CTB (0, 1, 2 and 4 μmol/L), Mdivi‐1 (50 μmol/L) or rapamycin (100 nmol/L) for 24 h. (A) HK activity was detected by Hexokinase Activity Detection Kit. (B‐C) Mitochondrial and cytosolic fractions were isolated. Western blot analysis showed the protein expression of HK2. (D) Representative fluorescence microscope images of SMMC‐7721 cells labelled with DAPI, HK2 antibody and Mito‐Tracker Green. Scale bar: 50 μm. (E) Glucose consumption was detected by glucose assay kit. (F) Production of lactic acid was assayed by Lactic Acid Production Detection kit. (G) ATP content was detected by the ATP Assess Kit. Data were presented as mean ± SD (n = 5); significance: *P < .05, **P < .01 and ***P < .001 vs control; ^#^ *P* < .05, ^##^ *P* < .01 and ^###^ *P* < .001vs CTB treatment

To investigate the potential interaction between autophagy, mitochondrial fission‐mediated mitophagy and glycolysis, we analysed the potential changes in glycolysis by introducing the autophagy activator rapamycin, which inhibits the mTOR (mammalian target of rapamycin) complex to activate autophagy. We first determine the dose of rapamycin, without affecting the survival of SMMC‐7721 cells (Figure S5A). We found that inhibition of hexokinase activity by CTB could be attenuated by Mdivi‐1, and rapamycin also significantly inhibited hexokinase activity (Figure 6A). The effect of CTB on the level of HK2 protein by autophagy allows us to determine whether it occurs at the transcriptional level or through protein stability. Rapamycin treatment had no significant effect on HK2 mRNA levels (Figure S5B). Therefore, autophagy did not affect HK2 at the transcriptional level.

In addition to the redistribution of HK2 from mitochondria to the cytoplasm, Mdivi‐1 weakened the inhibition of mitochondrial HK2 by CTB and rapamycin had a similar effect as CTB (Figure 6B, 6). Consistent with the above results, we demonstrated the effect of Drp1 activation and autophagy on HK2 distribution by immunofluorescence colocalization analysis (Figure 6D). As shown in Figure 6E and 6, we found that Mdivi‐1 had much milder effects on the glucose consumption and lactate production, weakening the inhibition of glycolytic activity by CTB. Interestingly, a significant reduction in glucose consumption and lactic acid production following rapamycin treatment was again observed. At the same time, similar conclusions were drawn from the investigation on the impact of ATP release (Figure 6G). Based on the above results, the influence of mitochondrial fission and CTB‐treated mitophagy on the aerobic glycolysis in cancer cells and specific regulatory mechanisms still need further exploration.

### 3.6 CTB showed tumour suppression effects in xenograft HCC model in vivo

**Figure 7:**
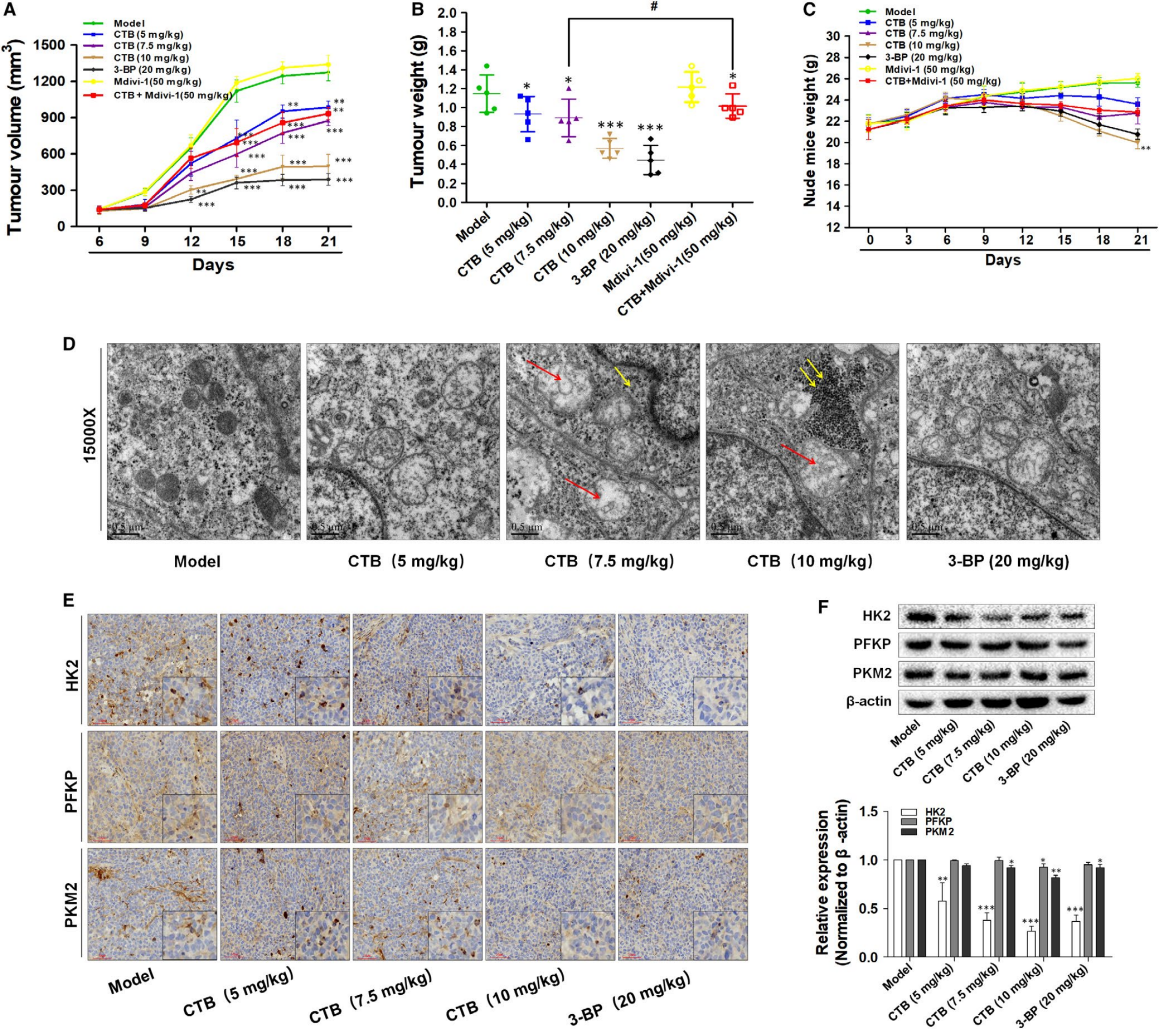
CTB inhibits the growth of HCC xenograft tumour in vivo. Nude mice with HCC cells xenograft were randomly divided into groups when tumour volume reached 150 mm^3^. (A) Tumour volumes were measured every three days and calculated using the formula: length × width^2^ × 0.5. (B) Tumour weights comparison, obtained on the final day of sacrifice in mice. (C) Body weights were recorded every three days. (D) Observation of tissues by transmission electron microscopy. swollen mitochondria (red arrow); accumulation of glycogen (yellow arrow). Scale bar: 0.5 μm. (E) Tumour tissues were subjected to immunohistochemistry staining with indicated antibodies to detect the change of HK2, PFKP and PKM2. Scale bar: 50 μm. (F) Western blot analysis showed the protein expression of HK2, PFKP and PKM2. Data were presented as mean ± SD (n = 8); significance: *P < .05, **P < .01 and ***P < .001 vs model; ^#^ *P* < .05, ^##^ *P* < .01 and ^###^ *P* < .001 vs CTB treatment

To validate the experimental results described above, we used HCC xenograft model established by subcutaneous inoculation of SMMC‐7721 cells in nude mice. Compared with vehicle (saline) alone, CTB or 3‐BP significantly substantially inhibited the tumour growth (Figure 7A, 7) and tumour mass (Figure S6A). In addition, CTB had no significant effect on mice weight at low and medium doses during the experiment. However, after 21 days of high‐dose treatment, CTB had a significant reduction in the final weight of the mice (Figure 7C), suggesting that was not significantly toxic to mice. These results preliminarily illustrated the inhibitory effect of CTB on tumour growth. The mitochondrial morphology was again observed in tumour tissues by TEM. Chromatin homogeneity, chromatin edges and mitochondrial matrix dispersion were observed in the model group. CTB promoted the disappearance of mitochondria and the reduction of matrix (Figure 7D). Mitochondrial swelling gradually became apparent (red arrow in Figure 7D), and glycogen accumulation can be seen in the cytoplasm (yellow arrow in Figure 7D). CTB significantly inhibited the accumulation of lactic acid, indicating a lower level of glycolysis (Figure S6B). In addition, the expression of HK2 was substantially decreased and the expression of PFKP and PKM2 slightly changed by immunohistochemistry and Western blot analysis (Figure 7E, 7). By inhibiting HK2 biological activity in tumour tissue, the energy supply to maintain tumour growth was blocked, and the proliferation of tumour cells was weakened.

**Figure 8:**
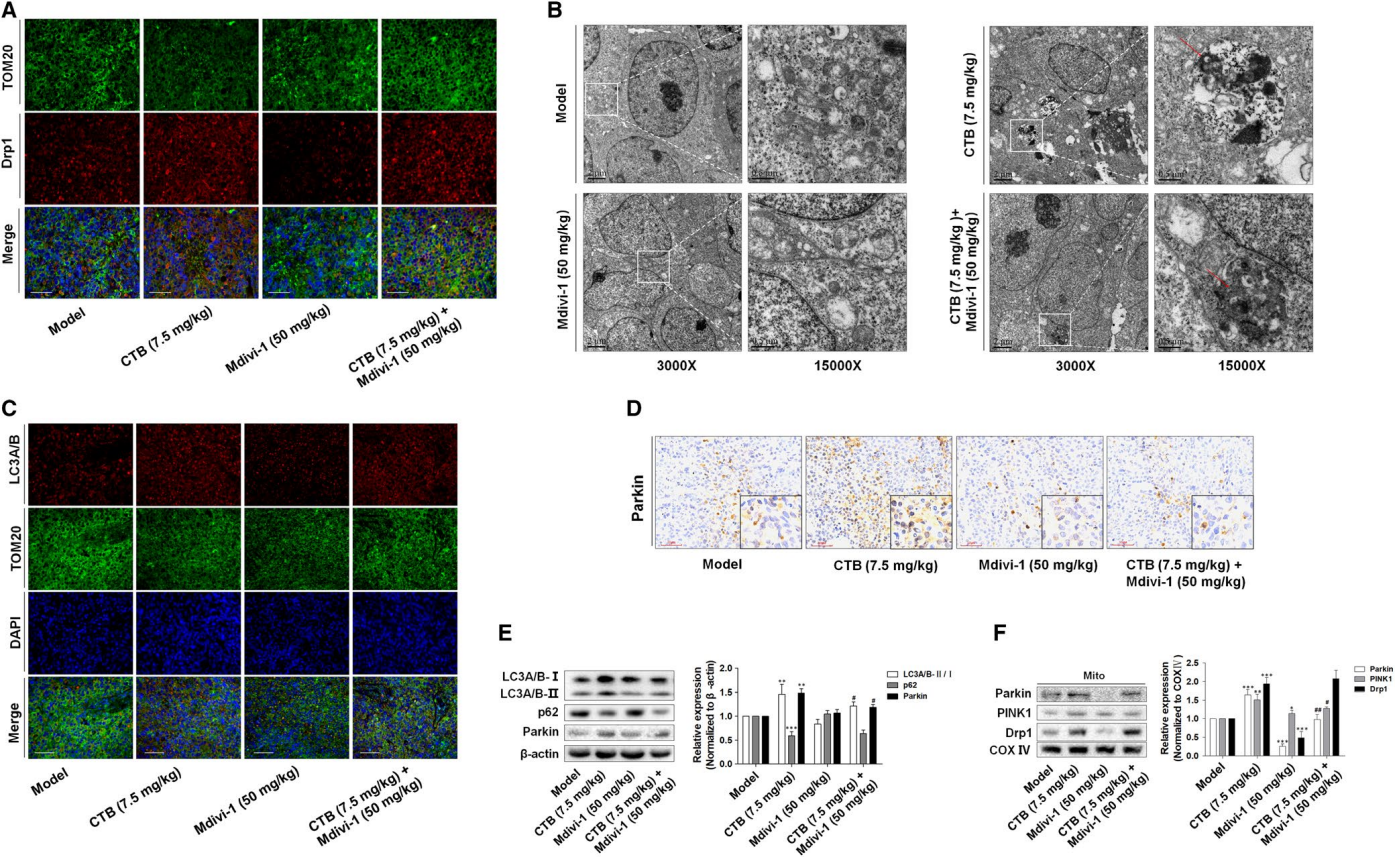
CTB‐regulated mitochondrial fission was closely related to the mitophagy in vivo. (A) Image of mitochondria and Drp1 colocalization were viewed with fluorescence microscope (Blue: DAPI; Green: TOM20; Red: Drp1). Scale bar: 100 μm. (B) Observation of mitophagy by transmission electron microscopy. Autophagosome (red arrow). Scale bar: 0.5 or 2 μm. (C) The detection of LC3A/B activation by fluorescence microscopy (Blue: DAPI; Green: TOM20; Red: LC3A/B). Scale bar: 100 μm. (D) Immunohistochemical analysis of Parkin in tumour tissues. Scale bar: 50 μm. (E) Western blot analysis showed the protein expression of LC3A/B, p62 and Parkin. (F) Western blot analysis showed the protein expression of Drp1, Parkin and PINK1 in mitochondrial debris of tumour tissue. Data were presented as mean ± SD (n = 8); significance: *P < .05, **P < .01 and ***P < .001 vs model; ^#^ *P* < .05, ^##^ *P* < .01 and ^###^ *P* < .001 vs CTB treatment

Evidence in vivo showed that CTB had a significant inhibitory effect on tumour growth in mice, but its role in targeting intratumoural mitochondria still require more validation. After treatment with Mdivi‐1, the elevated protein levels of Drp1 by CTB in rat fibrotic liver were decreased (Figure 8A). Autophagosomes are characterized by a vacuole‐like structure of a bilayer or multilayer membrane containing cytosolic components such as mitochondria, endoplasmic reticulum and ribosomes (Figure 8B). At the same time, we also detected LC3 and Parkin expression in tumour tissues and mitophagy showed high activation state, validating the results obtained in vitro (Figure 8C, 8). Consistently, the protein levels of LC3A/B‐Ⅱ, p62 and Parkin in tumour tissues were increased by CTB, and Mdivi‐1 weakened these effects (Figure 8E, 8), suggesting that Drp1‐mediated mitochondrial fission played a vital role in the initiation and development of mitophagy.

### 3.7 DISCUSSIONS

**Figure 9:**
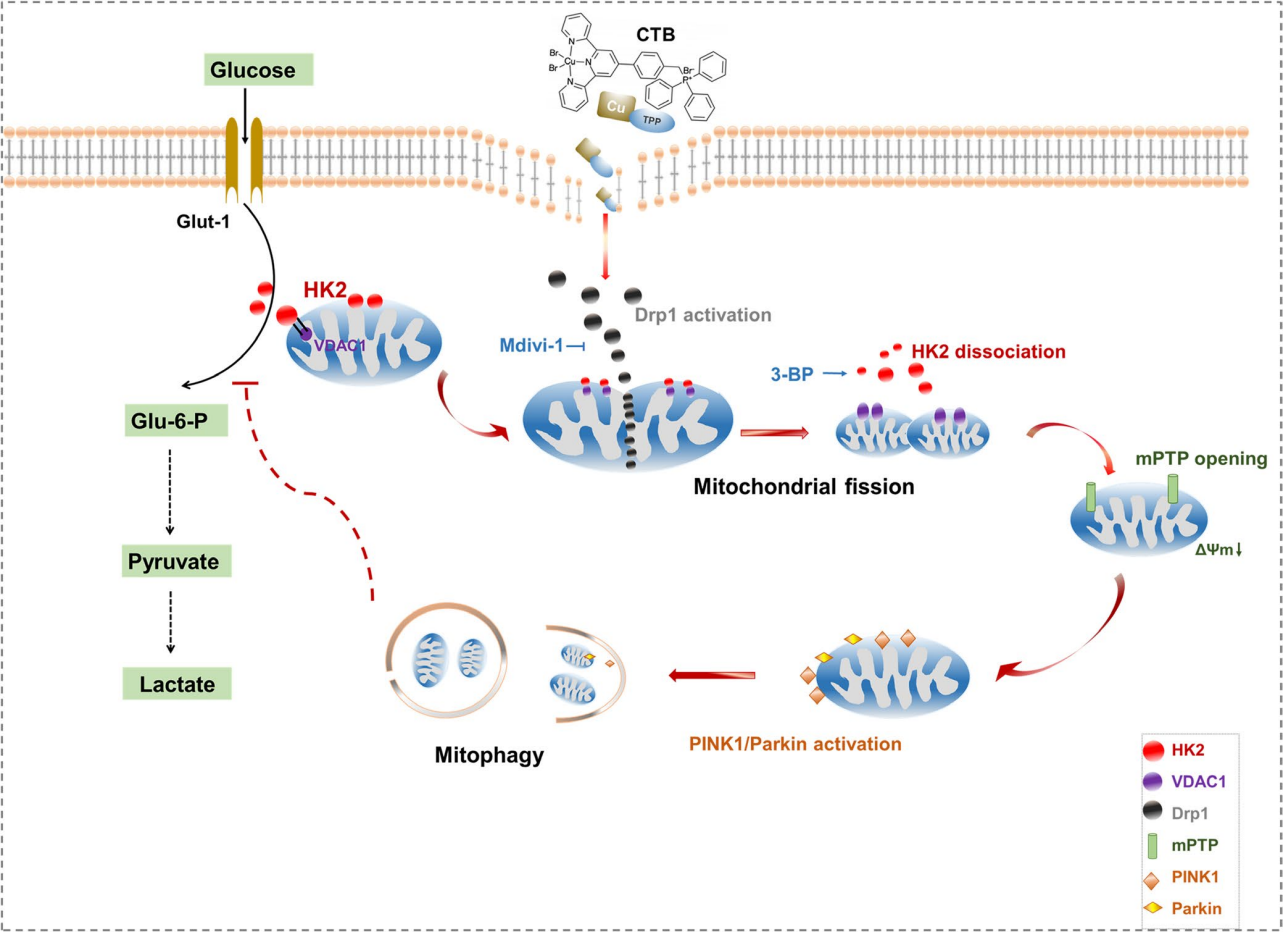
Schematic diagram of the damage mechanism of CTB on mitochondrial function and glycolysis in hepatoma cells

The physiological distribution, intracellular aggregation and inhibition of tumour cell growth of copper complexes have difference with platinum complexes, which opens up the potential for copper to become a potential anticancer agent.^38^ In recent years, the development of metal complex capable of targeting mitochondria for cancer treatment has increased, as the dysfunction of such organelles is associated with apoptosis and necrosis.^39^ In the synthesis of the new mitochondria‐targeted drug delivery system ZnPc/CPT‐TPPNPs, TPP can selectively accumulate hundreds of times in the mitochondria with good ROS response.^40^ In this study, TPP was introduced to the copper‐terpyridine complex in order to construct a novel anticancer complex. By investigating the molecular mechanisms by which CTB exerted anti‐tumour effects by targeting mitochondria, we discovered that CTB repressed glycolysis in HCC cells, and induced HK2 dissociation from the mitochondria via VDAC1 and mPTP opening. Lower ΔΨm and excessive mPTP opening activate the PINK1/Parkin ‐mitophagy pathway. CTB activates mitochondrial fission via Drp1, which is associated with mitophagy and glycolysis repression (See Figure 9).

HK2 has been shown to be overexpressed in many cancers and is considered a predictor of poor prognosis for HCC, breast and gastric cancer.^41^, ^42^ Many cancer studies have demonstrated the importance of HK2 in many cell life activities, including proliferation, metastasis and apoptosis.^43^ After CTB treatment, we found that lactate production and glucose consumption in HCC cells dramatically decreased. Interestingly, further researches revealed that there were differences in the expression of the key enzymes in the glycolysis process, showing HK2 played a vital role in glycolysis suppression. Since changes in glucose metabolism generally confer cancer cells resistance to chemotherapy, inhibition of glycolysis by targeting HK2 can significantly enhance the sensitivity of tumour cells to chemotherapy. Along with the alteration glucose metabolism often conferred cancer cells resistance to chemotherapy, and inhibition of glycolysis can significantly enhance the sensitivity of tumour cells to chemotherapy by targeting HK2.^44^ In the current study, we treated cells with CTB at 1, 2, 4 μmol/L, at which it could significantly act on mitochondrial HK2. HK2 has been shown to bind to the mitochondrial outer membrane via the oligomerization protein VDAC1. We provided evidence that CTB resulted in a weaker binding of HK2 to VDAC1, causing mitochondrial structure changes. It was known that mitochondria‐bound HK2 conferred anti‐cell death ability to cancer cells. Therefore, we considered that CTB inhibited glycolysis not only by reducing the overall HK2 activity in HCC cells, but also by focusing on changing its function on mitochondria.

From another perspective, we had found that CTB interrupted the bondage of HK2 to mitochondria. For further investigation, we used 3‐BP, inhibiting HK2 bound to VDAC1, and observed that CTB contributed to the opening of mPTP and the destruction of MMP in hepatoma cells. These findings suggested that the mitochondrial targeting group TPP, carried by the copper complex, significantly played a major role in affecting the structure of the mitochondria. We then observed the alterations of mitochondrial morphology after CTB treatment. Surprisingly, we detected the existence of mitophagy in CTB‐treated HCC cells. It has been demonstrated that abnormal destruction of mitochondria leads to the impairment of cellular aerobic respiration, resulting in massive energy depletion and eventual cell death.^45^, ^46^ The majority of damaged mitochondria are engulfed by lysosomes to proceed mitophagy, along with numerous dead cells. The dysregulation of mitophagy combined with cellular energy shortage, lessening the cellular resistance to various stress signals.^47^ We have found that CTB led to disorder of mitophagy in hepatoma cells. According to our current study, the main stimuli that promote mitophagy in hepatoma cells were the destruction of mitochondrial structure and the up‐regulation of PINK1/Parkin activation under CTB treatment. These processes may be the molecular mechanism by which mitophagy mediated cell death. As previously reported, the classical mitochondrial death pathway is characterized by excessive release of cytochrome C (Cyt C) from mitochondria into the cytoplasm. The mPTP opening is a necessary process for this type of death pathways.^48^ The opening of mPTP not only provides a channel for Cyt C leakage but also leads to the collapse of ΔΨm and attracts PINK/Parkin aggregation, which finally induces mitophagy.^49^ Our current results indicated that there was a close relationship between mPTP opening and mitophagy under CTB treatment in HCC.

Mitochondrial quality control is maintained dynamically by intracellular fusion and fission, and the imbalance of this process is closely related to tumour development.^17^ Undoubtedly, the occurrence of mitophagy inevitably mainly is related to the dynamics of mitochondria. Huang et al investigated the relationship between cytosolic calcium signalling and mitochondrial fission in HCC cells, showing that mitochondrial fission increased [Ca^2+^] concentration and calcium oscillation to promote global autophagy by the positive feedback loop.^50^ In the existing studies on copper element biological activity, it was found that after copper insult and accumulation, inhibition of PI3K/AKT/mTOR pathway activated autophagy and disrupted mitochondrial dynamics, forming positive feedback of redox barriers.^51^, ^52^ However, there are few studies on the relationship between mitophagy and mitochondrial dynamics, and the specific motivation and causal transformation have not been revealed. In our study, we aimed to investigate whether mitophagy was dependent on mitochondrial fission on the basis of the disruption of mitochondrial function by CTB. Under the induction of drugs, we observed a marked promotion of mitochondrial fission. Based on the original mitochondrial kinesis, the excessive fission had a certain auxiliary effect on mitophagy, and the inhibitor weakened the effects of CTB. Therefore, our results showed that mitochondrial fission involved in the process of mitophagy, and the effect of CTB on mitochondrial fission may play a crucial role in its anti‐tumour activity.

In conclusion, our group introduced TPP into the tripyridine complex to obtain a novel mitochondria‐targeted copper complex. Based on the preliminary determination of its anti‐tumour activity, we further confirmed how it affected mitochondrial function and better lay the foundation for the future development of tumour chemotherapeutics. CTB induced mitochondrial detachment of HK2, accompanied by changes in mitochondrial structure and activation of mitophagy, all of which were associated with mitochondrial over‐splitting. In turn, mitophagy, which was dependent on mitochondrial fission, also had a certain inhibitory effect on glycolysis. However, this study had some limitations. Further research is needed to support the direct impact of mitochondrial HK2 failure on glycolysis and to determine thresholds for excessive mitochondrial division. All in all, our current research leads us to believe that copper complexes with mitochondrial targeting potential inevitably bring about unique anticancer efficacy unreachably by other metal drugs.

## CONFLICT OF INTERESTS

The authors declare no potential conflicts of interest.

## AUTHORS CONTRIBUTIONS

ML and JS participated in the design of the study. ML performed cell culture work. ML, LW, YJ and FW performed animal study. ML and ZZZ performed biological testing. ML prepared the first draft of manuscript. ML and CJ performed the revised manuscript. XW, ZZ and FZ overviewed all data. ZG, JS and SZ supervised the whole study and reviewed the manuscript. All authors read and approved the final manuscript.

## Data Availability

Authors are willing to archive their data in a publicly accessible repository.
