# Supplementary material for: Novel mitochondrion‐targeting copper(II) complex induces HK2 malfunction and inhibits glycolysis via Drp1‐mediating mitophagy in HCC
Source: J Cell Mol Med. 2020 Jan 28;24(5):3091–107. doi: 10.1111/jcmm.14971 (PMC7077532; doi:10.1111/jcmm.14971)
Supplement: Supplementary file 1 [file JCMM-24-3091-s001.doc]

**
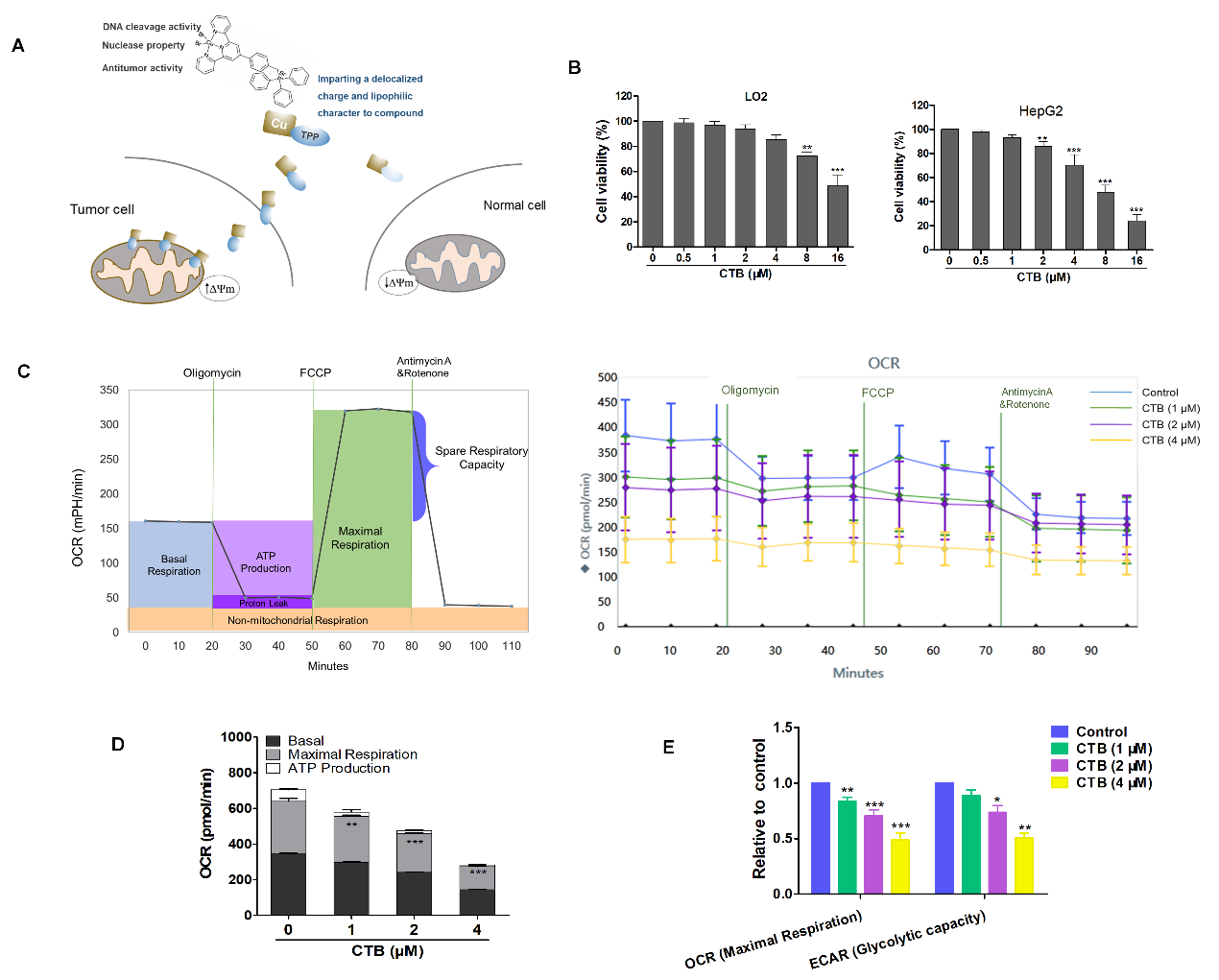
**

**Figure S1.** SMMC-7721 cells were treated with indicated concentrations of CTB (0, 1, 2 and 4 μΜ) for 24 h. **(A)** The illustration showed the targeting characteristics of CTB. **(B)** LO2 and HepG2 Cells viability was determined by the MTT assay. (**C**) Measurement of oxygen consumption ratio (OCR) using the XFe24 Extracellular Flux Analyzer. **(D)** OXPHOS variations (basal, maximal respiration, ATP production, and spare respiratory capacity) were summarized from raw data. **(E)** OCR(Maximal Respiration) and ECAR( Glycolytic capacity) levels of the above assessment were plotted as fold change relative to control by the Seahorse analyzer. Data were presented as mean ± S.D. (n = 5); Significance: *P < 0.05, **P < 0.01 and ***P < 0.001 vs control.


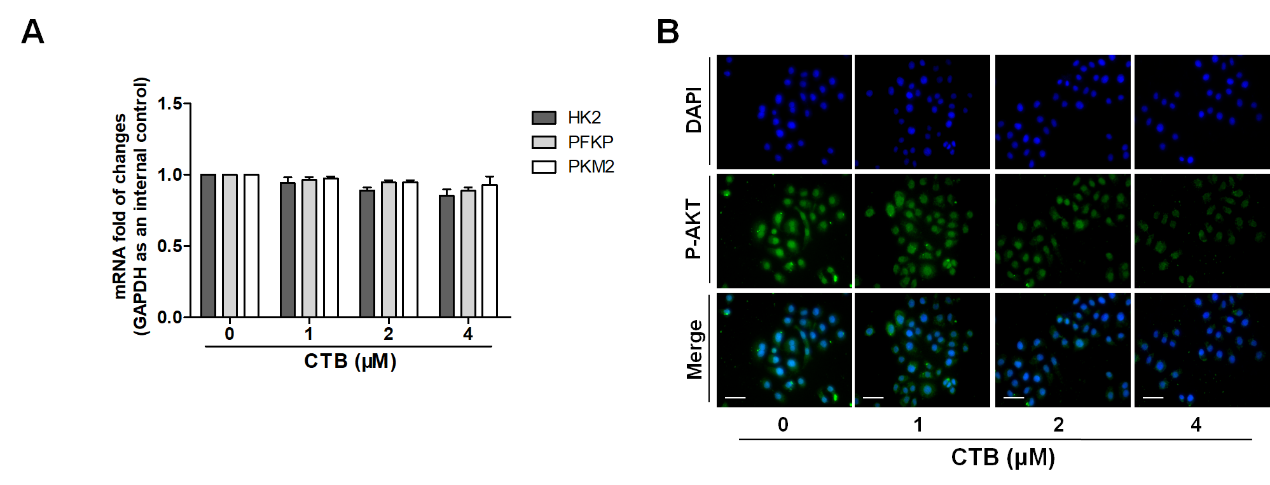


**Figure S2.** SMMC-7721 cells were treated with indicated concentrations of CTB (0, 1, 2 and 4 μΜ) for 24 h. (A) The levels of HK2, PFKP and PKM2 mRNA in the cells subjected to CTB were detected. (B) Immunostaining showed the activity of AKT. Scale bar: 100 μm. Data were presented as mean ± S.D. (n = 3); Significance: *P < 0.05, **P < 0.01 and ***P < 0.001 vs control.


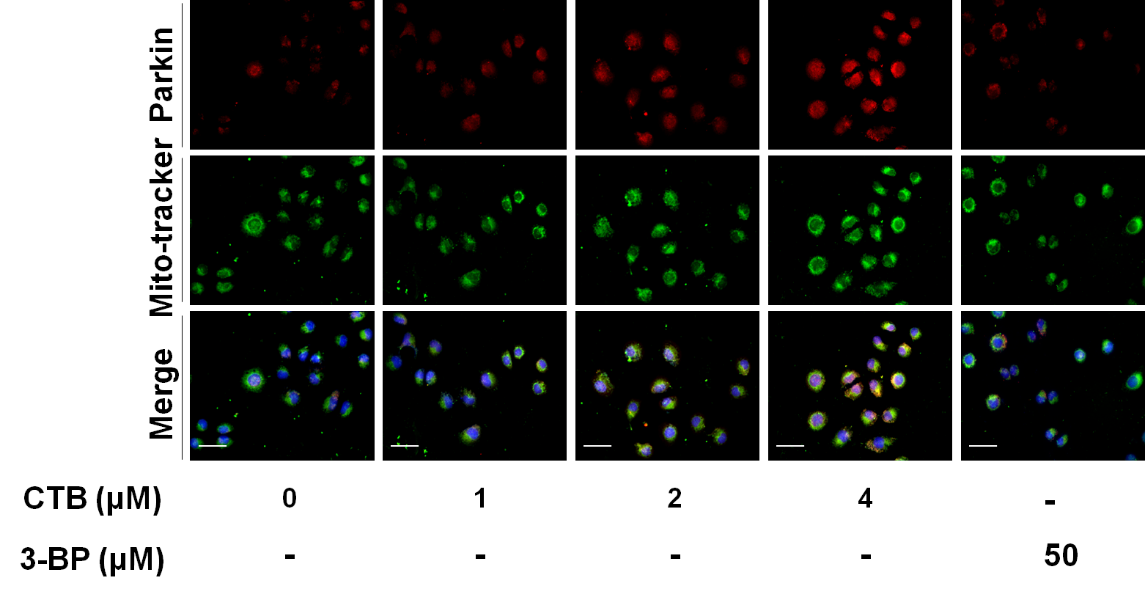


**Figure S3.** SMMC-7721 cells were treated with indicated concentrations of CTB (0, 1, 2 and 4 μΜ) or 3-BP (50 μΜ) for 24 h. The colocalization of Parkin and mitochondria were assessed by fluorescence microscopy. Scale bar: 50 μm. Data were presented as mean ± S.D. (n = 3); Significance: *P < 0.05, **P < 0.01, ***P < 0.001 vs control.


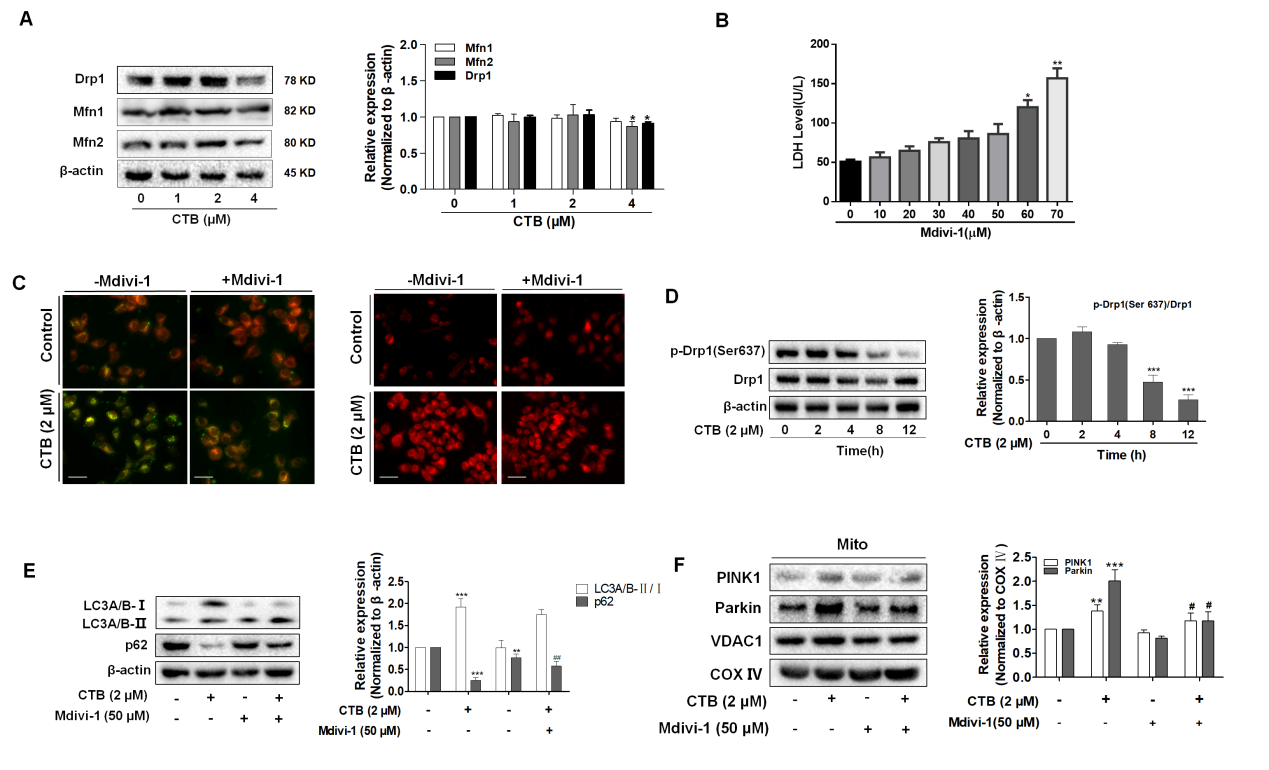


**Figure S4. (A)** SMMC-7721 cells were treated with indicated concentrations of CTB (0, 1, 2 and 4 μΜ) for 24 h. Western blot analysis showed the protein expression of Drp1, Mfn1 and Mfn2. **(B)** SMMC-7721 cells were treated with indicated concentrations of Mdivi-1 (10, 20, 30, 40, 50, 60 and 70 μΜ) for 24 h. LDH level was determined by kit. **(C)** The ∆Ψm was measured by JC-1 staining and mPTP opening was detected by Rhodamine 123 staining. Scale bar: 50 μm. **(D)** Protein levels of p-Drp1 and Drp1 were measured by Western Blot analysis. **(E)** Western blot analysis showed the protein expression of LC3A/B, p62 and parkin in SMMC-7721 cells. (**F**) Western blot analysis showed the protein expression of PINK1, Parkin and VDAC1 in mitochondrial protein extraction. Data were presented as mean ± S.D. (n = 3); Significance: *P < 0.05, **P < 0.01 and ***P < 0.001 vs control.


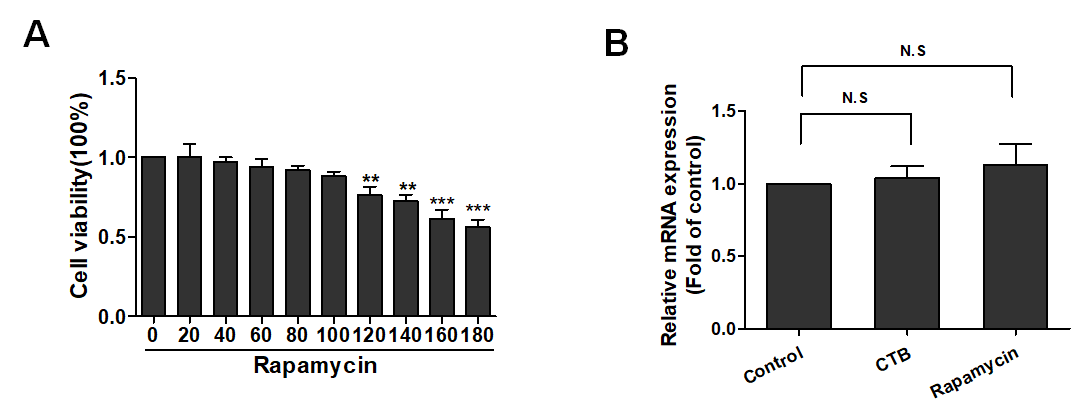


**Figure S5. (A)** SMMC-7721 cells were treated with indicated concentrations of Rapamycin (20, 40, 60, 80, 100, 120,140,160 and 180 μΜ) for 24 h. Cells viability was determined by the MTT assay. **(B)** SMMC-7721 cells were treated with indicated CTB (2 μΜ) and Rapamycin (100 μΜ). The levels of HK2 mRNA in the cells were detected. Data were presented as mean ± S.D. (n = 3); Significance: *P < 0.05, **P < 0.01 and ***P < 0.001 vs control.

**
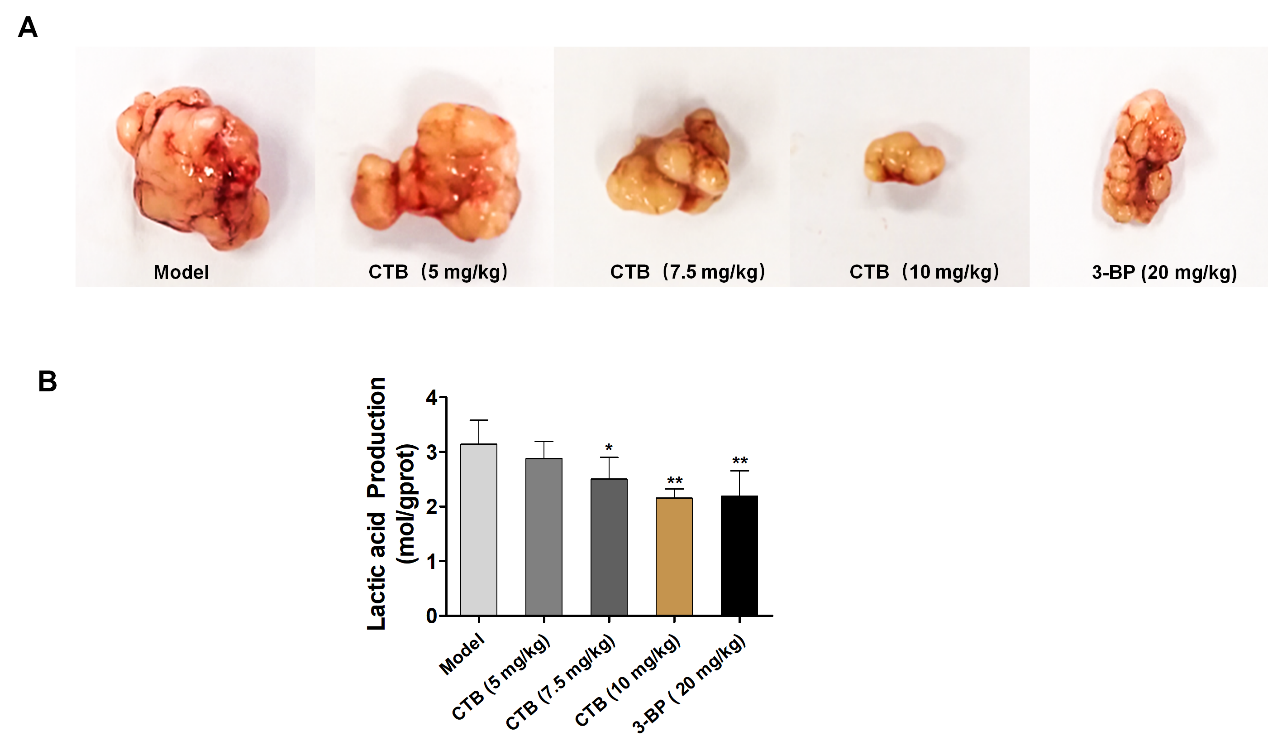
Figure S6.** CTB inhibited tumor growth and promoted apoptosis *in vivo*. **(A)** Representative images show the tumor xenografts after 21 days. **(B)** Levels of lactic acid was assayed by Lactic Acid Production Detection kit. Data were presented as mean ± S.D. (n = 3); Significance: *P < 0.05, **P < 0.01 and ***P < 0.001 vs control.
